# Evaluation of the Potential of High-Performance Liquid Chromatography–Inductively Coupled Plasma–Mass Spectrometry for the Determination of Chemical Warfare Agents and Their Toxic Degradation Products

**DOI:** 10.3390/molecules29215031

**Published:** 2024-10-24

**Authors:** Monika Kuligowska, Slawomir Neffe

**Affiliations:** Faculty of Advanced Technologies and Chemistry, Military University of Technology, Kaliskiego Street 2, 00-908 Warsaw, Poland; monika.kuligowska@wat.edu.pl

**Keywords:** HPLC-ICP-MS, chemical warfare agents, OPCW, verification

## Abstract

The determination of chemical warfare agents (CWAs) and their toxic degradation products (DPs) has become increasingly important for public and military safety in recent years. We focused on assessing the possibility of the HPLC-ICP-MS analytical technique to verify the provisions of the Chemical Weapons Convention. This technique enables the identification and determination of minimal concentrations (ppt range) of elements in various matrices. This fact is important for the determination of CWAs and other highly harmful compounds, even small amounts of which can have serious consequences for living organisms. We have critically analysed the results of scientific research on the identification and quantitative determination of extremely toxic organophosphorus, organosulfur and organoarsenic CWAs, their derivatives and their degradation products using high-performance liquid chromatography (HPLC) coupled with inductively coupled plasma–mass spectrometry (ICP-MS).

## 1. Introduction

The inductively coupled plasma mass spectrometry (ICP-MS) technique was designed in the early 1980s. Houk et al. [1] and Gray [2] published the first scientific research reports on the ICP-MS. Inductively coupled plasma was initially used for argon plasma ionization followed by optical emission spectroscopy (OES). ICP-OES allows for the determination of most metals and some nonmetals at the ppm to ppb level.

One of the disadvantages of ICP-OES is the use of measurement windows, which allow for the determination of only a few elements during a single scan, and a relatively high limit of detection (LOD). A breakthrough in the use of the ICP technique in chemical analysis was its coupling with a mass spectrometer. This made it possible to decrease the LOD of the analysis by three orders of magnitude compared with that of ICP-OES: from ppb to ppt. It also allows for an increase in the number of elements determined in a single scan analysis from several to even several dozen. Inductively coupled argon plasma with a quadrupole mass detector was combined with chromatographic separation techniques (GC-ICP-MS or HPLC-ICP-MS).

The HPLC-ICP-MS technique is not the first-choice technique for the determination of CWAs, but it is a promising tool that can be used to verify the identification of toxic chemicals [3].

The determination of CWAs and their degradation products using gas or liquid chromatography in conjunction with mass spectrometry has been extensively reviewed [4,5,6,7,8,9,10,11,12]. Compared with HPLC-ICP-MS, these methods have certain disadvantages. In the case of gas chromatography, degradation products from CWAs often require a time-consuming derivatization process at the sample preparation stage. Additionally, the complexity of the matrix complicates the analysis. For HPLC analyses, the use of atmospheric pressure chemical ionization (APCI) or electrospray ionization (ESI) to ionize a sample may deleteriously affect the mobile phase components and sample matrix components. In addition, the use of the APCI and ESI ionization modes is highly dependent on the analysed compounds. Therefore, expensive radiolabelled analogues are sometimes needed, which increases the analysis cost. The strong ionization used in the ICP technique is virtually independent of the matrix type. This feature enables the analysis of sample matrices that are considered too complicated for mass spectrometric analysis using ESI or APCI. An essential advantage of ICP-MS detection is the lack of complicated sample preparation procedures.

According to the research by Wilbur, the GC-ICP-MS technique enables rapid and sensitive qualitative and semiquantitative analyses of complex environmental samples containing unexpected analytes, including CWAs and organophosphate pesticides or compounds for which no analytical standards are available [13].

Following the contemporary security requirements for identifying CWA and other very toxic substances, four levels of identification are distinguished: first, presumptive; second, field confirmatory; third, theatre validation; and fourth, field definitive. The fourth level of identification requires the use of multiple established and independent protocols and technologies by scientific experts in internationally recognized laboratories to determine the unambiguous identity of a chemical hazard with the highest level of confidence and degree of certainty necessary to support strategic decisions [14]. We believe that chromatographic analytical techniques coupled with ICP-MS perfectly supplement the most widely used qualitative and quantitative analytical methods (for example, GC-MS/MS). The unambiguous identification of degradation products related to CWAs represents a cornerstone in the instrumental analysis plan established by the Organization for the Prohibition of Chemical Weapons (OPCW) and exercised by OPCW-certified laboratories around the world during yearly proficiency tests (PTs) [15]. The high polarity often associated with CWA degradation products (i.e., alcohols, amines, phosphonic acids and sulfonic acids) makes their detection by GC-MS particularly difficult because of their low volatility. As most of the preliminary methods involve GC-MS and related combined methods, the derivatization of these dialkylethanolamines is necessary during the execution of these analyses. Derivatization of analytes results in their conversion into more volatile species (i.e., lessened polarity) that exhibit chromatographic profiles (i.e., peak shapes and different retention times) different from those of the original analytes, which have not been subjected to derivatization.

This review describes the possibilities of the ICP-MS technique coupled mainly with high-performance liquid chromatography (HPLC) in the analysis of samples potentially containing CWAs, their derivatives, and products of their degradation, as well as some other highly toxic substances. Notably, HPLC-ICP-MS is a complementary technique to GC-MS and LC-MS, but only for CWAs that contain phosphorus, sulfur or arsenic atoms in their molecules. On the other hand, CWAs that contain nitrogen and oxygen atoms [e.g., bis(2-chloroethyl)ethylamine), bis(2-chloroethyl)methylamine and/or tris(2-chloroethyl)amine] cannot be selectively determined by HPLC-ICP-MS.

## 2. HPLC-ICP-MS in the Determination of Organophosphorus Compounds

### 2.1. The Importance of Determining Organophosphorus Nerve Agents, Their Hydrolysis Products, and Their Metabolites

Organophosphorus nerve agents (OPNAs) are the most toxic and deadliest compounds. These electrophilic compounds react with the nucleophilic serine residue in the active site of acetylcholinesterase (AChE), the enzyme responsible for the breakdown of the neurotransmitter acetylcholine (ACh). By blocking AChE, organophosphate chemical warfare agents paralyze the nervous system [16]. Although nerve agents were used before World War II, they have been continuously used in modern times [17,18]. The first recorded attack occurred on 15 March 1988, when Saddam Hussein’s troops used sarin in Halabja during the Iran–Iraq war [19,20]. Approximately 5000 Kurdish citizens died then. One of the most shocking incidents in the world, in which organophosphorus warfare was used was the terrorist attack by the Aum Shinrikyo sect (Supreme Truth) on the Tokyo subway on 20 March 1995 [21,22]. Fourteen people were killed, but if the concentration of sarin had been distributed differently, the death toll could have been much higher. There is undeniable evidence that sarin was used on a large scale in Syria between 2013 and 2018 [23]. Kim Jong Nam, the half-brother of Kim Jong Un, the leader of North Korea, died of poisoning with the VX agent. The incident took place at Kuala Lumpur Airport in February 2017 [24,25]. Attacks with a little known nerve agent took place in Salisbury in 2018 [26,27]. The detected substance was a compound with the code A-234, belonging to a group of compounds known as Novichoks. Sergei Skripal and his daughter, Yulia, were first poisoned with this agent, followed three months later by two British citizens, Charlie Rowley and Dawn Sturgess [28].

### 2.2. Hydrolysis of Organophosphorus Nerve Agents

**Figure 1:**
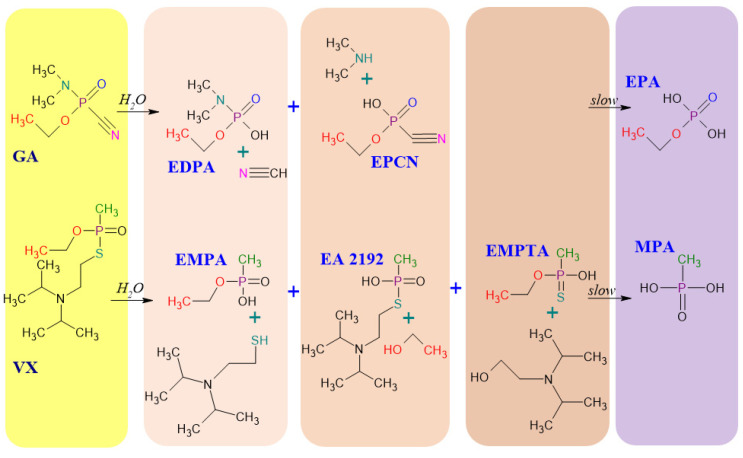
Simplified scheme of possible hydrolysis pathways for tabun (GA) and VX [5,30].

**Figure 2:**
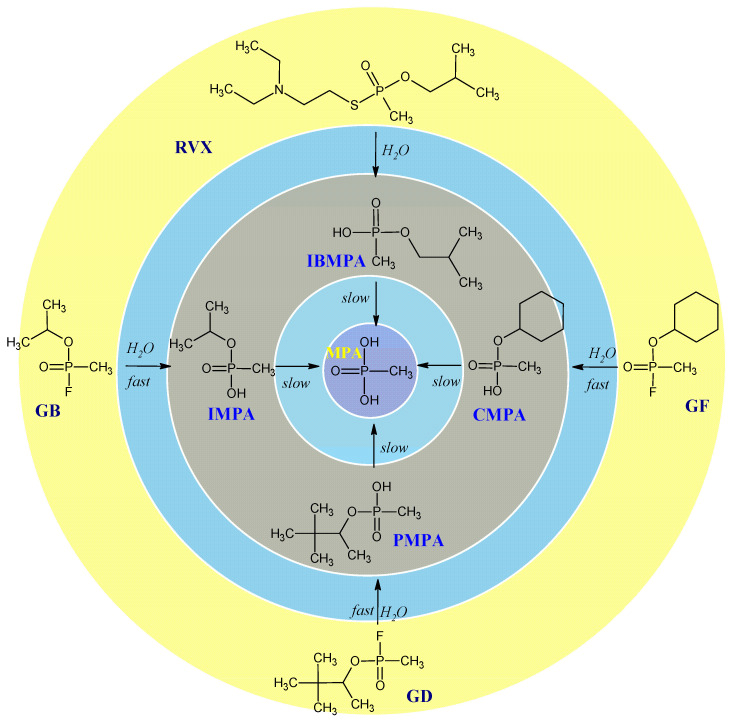
Simplified hydrolysis scheme for RVX (S-[2-(diethylamino)ethyl] O-(2-methylpropyl) methylphosphonothioate; known as Russian VX), sarin (GB), soman (GD), and cyclosarin (GF). Starting substances (CWAs) are marked on a yellow background, intermediates are marked on a beige background, and the product of the hydrolysis of these compounds (MPA) is marked on a purple background [5,31,32].

To illustrate the structure and behaviour of organophosphorus nerve agents under environmental conditions, Figure 1 shows the possible pathways of the hydrolysis of ethyl N,N-dimethylphosphoramidocyanidate, popularly called tabun, in military terminology marked as GA and S-{2-[Di(propan-2-yl)amino]ethyl} O-ethyl methylphosphonothioate known as VX. The product of GA degradation is ethylphosphoric acid (EPA), while the product of VX hydrolysis is methylphosphonic acid (MPA). The VX hydrolysis leads to several intermediates (EMPA, EA 2192, EMPTA) [29]. Degradation reactions of other OPNAs, leading to the final product, which is MPA, are presented in Figure 2. Notably, the degradation products of organophosphorus CWAs are highly polar and low-volatile. However, OPNA degradation products (OPNA DPs) have almost equal toxicity to that of the initial agents, so their determination may be an excellent alternative for researching parent compounds. Moreover, the presence of specific degradation products of OPNA chemical warfare agents in the environment indicates, with high probability, the use of these extremely toxic agents.

### 2.3. The Use of the HPLC-ICP-MS Technique for the Determination of Organophosphorus Compounds

Because of its very low detection limit (LOD) at the ppt concentration and good separation of analytes, the HPLC-ICP-MS technique is beneficial for analysing trace concentrations of OPNAs and their degradation products. Initially, the application of HPLC-ICP-MS for the determination of organophosphorus compounds was not successful due to the high potential of the first ionization of the ^31^P^+^ ion, which is 10.5 eV, as well as the interferences caused by the presence of ^14^N^16^O^1^H^+^ and ^15^N^16^O^+^ ions (*m*/*z* 31) [31].

However, using a special system in the ICP-MS apparatus, which was part of the collision/reaction cell, the interferences of the so-called octopole reaction system with helium as the collision gas were eliminated, as a result of which the background signal was reduced.

The first work on the determination of OPNA degradation products using gas chromatography combined with ICP-MS was published by the research group led by D.D. Richardson and J.A. Caruso [31]. Their research optimized and validated a method that allowed for the separation and determination of seven organophosphorus compounds, namely EMPA, IMPA, EDPA, IBMPA, PMPA, MPA, and CMPA, in less than 10 min, with a detection limit of less than 50 pg/mL. This method has been successfully implemented for the analysis of river water and soil samples. The interfering polyatomic species ^14^N^16^O^1^H^+^ and ^15^N^16^O^+^ were mainly produced in a plasma torch operating at atmospheric pressure, so they were only partially related to the system used for the introduction of the sample. In the case of gaseous sample injection used in gas chromatography, interferences of atmospheric origin were lower than in the case of analyses conducted using liquid chromatography. This resulted in a lower background level for the *m*/*z* 31 signal. As stated in the research by Richardson and Caruso, the background level obtained was so low that the use of a collision reaction cell to improve this parameter was unnecessary. However, the use of He in the collision reaction cell was mandatory for obtaining a negligible background in the mass window. Because of the best stability of the obtained products, TBDMS was used for the derivatization of the tested compounds. The derivatization reaction conditions were optimized and run at 80 °C for 45 min. The derivatization process, which was necessary to obtain more volatile compounds for gas chromatography analysis, increased the sample preparation time. Valdez and Leif in a review article on the application of the derivatization of OPNA degradation products for their determination in environmental samples by GC-MS [32] described the processes of silylation using N,O-bis(trimethylsilyl)trifluoroacetamide (BSTFA) and N-tetrbutyldimethylsilyl-N-methyltrifluproacetamide (MTBSTFA) and methylation with diazomethane. They indicated that new derivatizing reagents were needed to enable the detection of OPNA’s DPs by GC-MS at a level of <1 ppm.

The studies carried out by the research team of J.A. Caruso optimized the method of determining organophosphorus chemical warfare agents using the ICP-MS technique combined with liquid chromatography [33]. Research showed that the use of gas chromatography, in this case, reduced the detection limit by 64 to 89% while maintaining the correct separation of compounds. The results obtained for both studies are summarized in Table 1. However, it should be noted that despite the better LOD of the results obtained, when GC-ICP-MS was used, the determination of phosphonic acids was burdened by the extension of time for sample preparation procedures (caused by derivatization).

The determination of the OPNA DPs is challenging in food samples because some food products my contain significant concentrations of phosphoric acid (V), which is used in the food industry under the designation E338. Phosphates can also be found in food of plant origin because they are used to produce fertilizers in agriculture. Two papers from the J.A. Caruso research group described the method of OPNA DP determination in samples containing high concentrations of inorganic phosphates [34,35]. The research objects for the study included samples of food products available in stores, such as bottled water, fruit juice, infant formula, isotonic beverages, and lettuce. In this study, three methods were investigated to separate the compounds used as the target of research. One method used a Dionex IonPac AS7 column (Thermo Fisher Scientific, Waltham, MA, USA) with a precolumn, and the other two methods used a Hamilton PRP-X100 column (Hamilton Company, Boston, MA, USA). Some parameters of the separation techniques used in the study are summarized in Table 2.

The technique of analyte separation using the Dionex IonPac AS7 column was used for the separation of VX and sarin degradation products, EMPA and IMPA, in the presence of MPA and H_3_PO_4_ using LC-ICP-MS. This method provided a satisfactory baseline. The separation and identification of all these compounds took less than 5 min. Separation techniques using the Hamilton PRP-X100 column resulted in an increase in analysis time. When the number of analytes (OPNA DPs) increased, the peaks of IPHEP with MPA and IBHMP with H_3_PO_4_ overlapped. The research allowed for the separation of ten OPNA degradation products within 25 min. This technique, which combines the Hamilton PRP-X100 column with an ammonium carbonate solution elution system, was successfully implemented in determining OPNAs with a structure like MPA and a pKa value similar to the pKa value of H_3_PO_4_. The analysis was based not only on ICP ionization but also electrospray ionization (ESI). To carry out analyses identifying ^31^P^+^ ions, an ICP-MS system equipped with a collision reaction cell, with helium as the reaction gas, was used. To maintain the stability of the signal from the *m*/*z* 31 ion and reduce the noise level, the helium flow rate was optimized to 2 mL/min. Electrospray ionization was performed in positive mode. The technique implemented was compatible with LC-ICP-MS and LC-ESI-MS analyses if the buffer concentration was relatively low, resulting in good separation of seven OPNA DPs in less than 30 min. The compatibility of the techniques allows for the identification and verification of the structure of OPNA DP derivatives. The studies showed that both techniques, LC-ICP-MS and LC-ESI-MS, were characterized by similar LOD values [34,35].

## 3. ICP-MS in the Determination of Organosulfur Compounds

### 3.1. Importance of Organosulfur Compound Determination

The ICP-MS technique was used to analyse not only OPNAs but also necrotic chemical warfare agents and their derivatives, and metabolites in different samples. 1-Chloro-2-[(2-chloroethyl)sulfanyl]ethane—sulfur mustard (HD)—is one of the best-known chemical warfare agents [36]. This so-called mustard gas was used for the first time during World War I near Ypres [36,37]. This agent was also used later, for example, in China in 1937–1945 and during the Iran–Iraq war in 1983–1988 [38,39,40]. Currently, the greatest threat is posed by the remains of chemical weapons, which have been dumped, among others, in the Baltic Sea after World War II. At the bottom of this sea, approximately 15,000 tons of poisonous warfare agents were dumped, 63% of which were sulfur mustard, which filled barrels, artillery shells and bombs [41,42,43].

### 3.2. Degradation Reactions of Organosulfur Compounds

**Figure 3:**
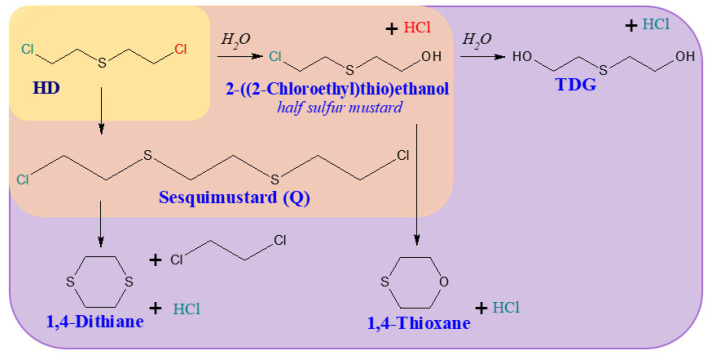
The most common 1-chloro-2-[(2-chloroethyl)sulfanyl]ethane degradation products [48].

Among the organosulfur compounds, the most dangerous from the view point of use on the battlefield is 1-chloro-2-[(2-chloroethyl)sulfanyl]ethane. It is a type of vesicant chemical warfare agent, but also causes intense general poisoning effects [44,45]. Sulfur mustard is readily hydrolysed in water, forming 2-((2-chloroethyl)thio)ethanol (known as half-sulfur mustard). The main product of the complete hydrolysis of HD is thiodiglycol (TDG). Other products of sulfur mustard degradation may include 1,4-thioxane and 1,4-dithiane [46,47]. However, the number of degradation products may include more than 20 additional compounds [48,49]. The hydrolysis of sulfur mustard and the main degradation products are shown in Figure 3.

### 3.3. Use of the ICP-MS Technique to Determine Organosulfur Compounds

Several research works have described methods of the determination of organosulfur toxic agents, for example, using gas chromatography coupled with mass spectrometry and other techniques [50,51]. In the work of Kroening et al., five sulfur mustard hydrolysis products were determined using the HPLC-ICP-MS technique [52]. For comparison, HPLC-ESI-MS was used in positive ion mode. The analyses were carried out using a single and tandem mass spectrometer (ESI-MS and ESI-MS/MS).

This was the first work to describe the determination of sulfur mustard degradation products using HPLC-ICP-MS, which was equipped with a collision cell module [52]. One of the significant advantages of this technique was the application of a collision cell with collision gas, i.e., xenon, which allowed for the elimination of interference from the ^32^S^+^ channel, for example, from ^16^O^16^O^+^ and ^14^N^18^O^+^ ions. The optimal gas flow for these analyses was 0.25 mL/min. The most considerable polyatomic interferences were minimized by matching the appropriate voltage to the octopole (−46 V) and quadrupole (−1 V). A positive upstream quadrupole voltage of +45 V resulted in the selective transmission of ^32^S^+^ while minimizing the larger positive ion polyatomic interferences. In this study, displacement chromatography was used to improve the analysis parameters. 2-Methyl-3-pentanol, 3-pentanol, and 2,2-dimethyl-3-pentanol were used as displacers [52]. The results were verified using different column types and displacement coefficients. The considered methods were successfully applied for the analysis of river water samples. The selected parameters of these determinations are shown in Table 3.

## 4. ICP-MS for the Determination of Organoarsenic Compounds

### 4.1. Significance of the Determination of Organoarsenic Compounds

Organoarsenic CWAs were used during World War I, constituting a kind of “replacement” for sulfur mustard [36]. These include Lewisite, diphenylchloroarsine DPCA (Clark I or DA), diphenylcyanoarsine DPCNA (Clark II or DC), phenylodichloroarsine PDCA (Pfiffikus) and 10-chloro-5,10-dihydrophenazarsinine (Adamsite). Many ammunitions containing these agents were dumped in the Baltic Sea [53,54] and were found on seashores in Belgium, Germany, Denmark and Poland. Also, in China and Japan, the presence of degradation products of organoarsenic CWAs was found in the soil [55] and groundwater [56]. During World War II, organoarsenic CWAs were produced in Japan, for example, on Okuno Island in the Seto Inland Sea. After World War II, some produced organoarsenic agents were solidified in concrete and illegally buried, among others, in closed gravel pits. To this day, there are cases of poisoning, such as, for example, workers contaminated during road construction in the cities of Samukawa and Hiratsuka, Kanagawa Prefecture, or the city of Kamisu, Ibraki Prefecture [57,58].

The determination of organoarsenic CWAs in environmental samples is difficult, especially in industrialized areas and the marine environment. In such cases, speciation analysis plays an important role in determining the origin of arsenic compounds. When searching for residues of organoarsenic CWAs in the environment, the probability of finding several types of degradation products is high. Biological samples may contain arsenobetaine (AB), which occurs naturally in living organisms, especially marine organisms, and human urine

### 4.2. Degradation Reactions of Organoarsenic Compounds

**Figure 4:**
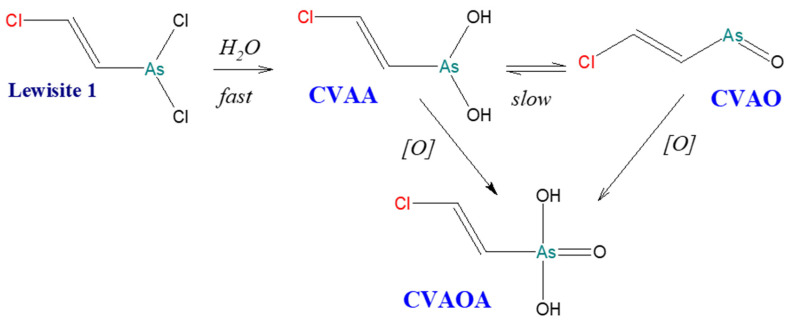
Hydrolysis of [(E)-2-chloroethen-1-yl]arsonous dichloride (Lewisite 1) [5].

**Figure 5:**
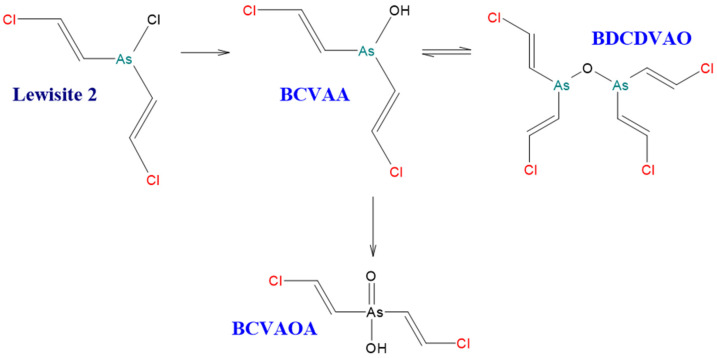
Hydrolysis of bis[(E)-2-chloroethen-1-yl]arsinous chloride (Lewisite 2) [5].

**Figure 6:**
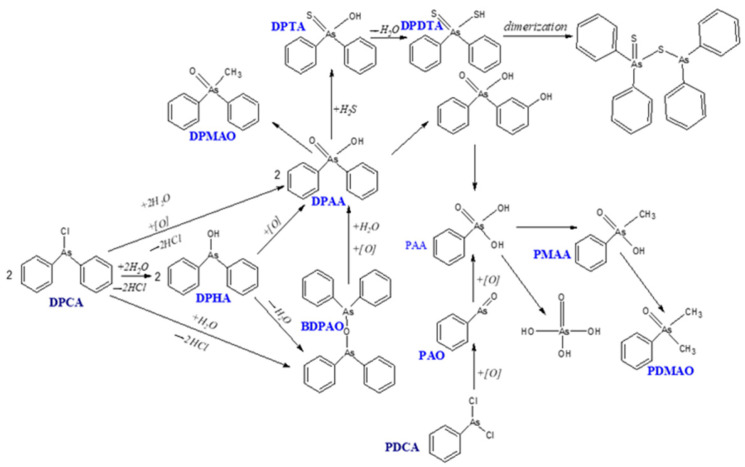
Scheme of possible degradation pathway reactions of DPCA and PDCA [59,60].

Figure 4 and Figure 5 show diagrams of Lewisite 1 and Lewisite 2 hydrolysis. Figure 6 shows a diagram of the DPCA (DA) hydrolysis reaction and possible degradation products. A significant number of the same products are formed during the environmental degradation of the DPCNA.

### 4.3. The Use of the ICP-MS Technique for the Determination of Organoarsenic Compounds

The determination of arsenic compounds in biological and environmental samples is critical because the mechanism of metabolic transformation of these compounds in living organisms and redox reactions in the environment are still not fully understood.

GC combined with mass spectrometry is an excellent tool for the determination of organoarsenic CWAs. Unfortunately, low-volatile arsenic compounds, such as DPAA, require prior derivatization with, e.g., 1-propanethiol or thioglycolic acid methyl ester. Derivatives have an unpleasant odour and are unstable at elevated temperatures, which may cause their thermal decomposition in the injection port of the GC. This can cause problems with the accuracy and reliability of the obtained results. For this reason, the determination of organoarsenic compounds by HPLC-ICP-MS is a good alternative to GC-MS. ICP-MS also has some limitations. Interference from other ions is the most significant problem. Chlorine may be present in a test sample, especially in environmental samples. In this case, the ion ^40^Ar^35^Cl^+^ can be produced in the plasma with an *m*/*z* ratio of 75, the same as the only stable isotope of arsenic, ^75^As^+^. Next, although much less likely, interferences may be caused by ions ^150^Nd^2+^ and ^150^Sm^2+^, which also have *m*/*z* 75 [61]. However, they are rare in environmental samples. When a single quadrupole is used for analysis, the chlorine ions (ArCl^+^) formed in the plasma can be eliminated using a collision cell module with helium. Another possible solution is to use a double quadrupole, which removes interference from ArCl^+^, Nd^2+^ and Sm^2+^ ions. When triple quadrupole is used, an ion with *m*/*z* 75 is isolated in the first quadrupole (Q1). It is then transported to an oxygen collision reaction cell, where the ^75^As^16^O^+^ ion with *m*/*z* 91 is formed, which is isolated in the second quadrupole (Q2) and then transmitted to the MS detector. When the *m*/*z* 91 ion is monitored, no *m*/*z* 75 ions are detected, so this is a reliable way to eliminate these interferences. The use of high-resolution mass spectrometers can overcome these limitations [62].

Difficulties in HPLC-ICP-MS analysis may occur when analysing solutions with extreme pH values. Such samples may cause shifts in the retention times of analytes or contribute to the precipitation of phosphates from the mobile phase of the HPLC column. However, this risk can be avoided by using an appropriate sample preparation method. Thus, as discussed previously, the potential limitations of the technique can be eliminated. Analyses of organoarsenic compounds carried out using the ICP-MS technique have achieved an LOD in the ppt range and an excellent stability of the results. An additional benefit is the shortening of the analysis time because the sample preparation procedure is quick and straightforward, and the analysis, even of multicomponent mixtures, takes a few to several minutes. Some examples of the use of this technique for determining organoarsenic compounds are given below.

In 2003, a group of scientists led by Ishizaki conducted the first study of drinking water from the Kizaki area of Kamisu, Ibaraki Prefecture, in Japan [57]. This research was carried out due to specific symptoms impairing the central nervous system in humans and the high mortality rate among animals living in these areas. Researchers were surprised that the patient’s health improved after leaving the area where they lived for an extended period and deteriorated a few days after returning to the city. Water samples from the taps of a multifamily house were analysed. Water was extracted from a depth of approx. 12 m from a well dug in the reclaimed area of a former gravel pit. The first analyses were performed using GF-AAS. Research showed that the cause of health disorders were arsenic compounds, the content of which in drinking water exceeded the permissible concentration (0.01 mg/dm^3^) by a factor of 450. Moreover, the other parameters that drinking water should meet, such as the contents of heavy metals, volatile organic halogens, or organophosphorus and chlorine pesticides in the collected samples, were within the normal range.

Studies revealed that the concentration of DPAA in drinking water samples was a maximum of 15 ppm, which meant that the inhabitants of this city could consume up to 30 mg of DPAA per day [57]. GF-AAS analysis revealed that the content of inorganic arsenic was 1.5 ppm, and the total concentration of this element was 4.5 ppm. This means that the concentration of organic arsenic was 3 ppm. However, using HPLC-ICP-MS, it has been proven that arsenic contamination of drinking water comes entirely from the organic form of this element, i.e., compounds such as BDPAO, DPAA, and PAA, and that this contamination was the result of the environmental degradation of organoarsenic warfare agents: diphenylchloroarsine (DPCA) and diphenylcyanoarsine (DPCNA).

The content of inorganic arsenic in previous analyses (conducted with the GFAAS technique) was caused by the inaccurate process of sample preparation for analysis and the ingress of phenolic arsenic compounds into the aqueous phase during extraction. A thorough analysis using the ICP-MS technique enabled the error to be detected and the correct results to be obtained. Due to the severity of the threat, more studies of drinking water from the region have been conducted. Kinoshita et al. developed a method for the simultaneous determination of DPAA and PAA in drinking water using HPLC-ICP-MS to detect contamination quickly [58]. Water samples were taken from six wells at depths of approximately 5–6 m in the same area—the city of Kizaki in Japan.

The samples were passed through a membrane filter with a diameter of 0.45 μm and stored in amber glass, polypropylene or polyethylene vials to prevent the contamination of the samples with trace amounts of arsenic contained in low-quality glass. The mobile phase consisting of acetonitrile and water (30:70) at pH = 2 was used for HPLC analyses, and each injection had a volume of 5 µL. By using the proposed method, the arsenic compounds were separated within 4 min, reaching the detection limits for DPAA (2 ng/mL) and PAA (1 ng/mL).

The calibration plots were characterized by excellent linearity, and the method was characterized by good reproducibility (RSD = 1.7–6%). In addition, long-term analyses were also performed, and the arsenic determination revealed stability even after 7 h (RDS = 1.3–5.8%). However, it was not entirely clear whether the PAA contained in the samples came from the environmental decomposition of DPAA, i.e., whether it was a product of diphenylchloroarsine and diphenylcyanoarsine (DPCA and DPCNA) decomposition, or whether it was a product of phenyldichloroarsine degradation. The optimized method, which yielded excellent results, allowed its ability to be adapted for the analysis of phenylarsenic compounds on a large scale. To extend the research and refine the method, the team of Kinoshita et al. validated the HPLC-ICP-MS method for analysing organoarsenic compounds, including Lewisite 1 degradation products [63].

Because chlorine is often present in biological samples, signal measurements were performed considering the AsO^+^ ion (*m*/*z* 91). Because of this, the signal from the ArCl^+^ ion, which was the most intense source of interference, did not cross the baseline. A dynamic reaction cell (DRC) module and oxygen were used as the collision reaction gas. Some urine samples were collected from mice orally exposed to the CVAA standard solution—the main decomposition product of Lewisite 1, which is also a toxic compound. Samples were taken after 24 h. The analytes were CVAA and the main degradation metabolite of this compound, i.e., CVAOA. For greater accuracy of the tests, several sample HPLC columns were verified: Inertsil C4, C8, and Ph (2.1 × 150 mm, 5 μm); and CHEMCOSORB 3-Dph (2.1 × 150 mm, 3 μm). These columns are typically used for the determination of phenyl compounds. The best separation of compounds was obtained using the C8 column, and therefore it was used for further studies. The injection volume was also verified. A series of studies led the authors to the conclusion that with the increase in the injection volume, the detection limit decreased. The injection volume was therefore set at 20 μL. The developed method was successfully implemented to analyse human urine samples contaminated with degradation products of organoarsenic warfare agents: CVAA and CVAOA in the presence of DPAA and PAA. The analysis was characterized by a low LOD and a short duration.

All the determined compounds were well separated within 4 min. DPAA was identified only after 12 min. The detection limits were as follows: CVAA 0.2 ng As/mL, CVAOA 0.1 ng As/mL, DPAA 0.5 ng As/mL, and PAA 0.3 ng As/mL. Urine analysis of CVAA-treated mice revealed the presence of several unknown compounds that are analyte metabolites. The method proposed in this paper enables the speciation analysis of arsenic. Additionally, it provides valuable information that cannot be obtained by determining the total arsenic content of a sample or by procedures involving derivatization as one of the sample preparation steps. This was confirmed by the fact that the HPLC-ICP-MS technique can be successfully implemented in research on the metabolism of organoarsenic compounds in living organisms. Because of reports of methylated degradation products of phenylarsine warfare agents detected in environmental samples in Japan, further research involving HPLC-ICP-MS analysis methods was needed. The research development on this topic led to another work by the Kinoshita team. They described methods for the determination of organoarsenic compounds in urine using HPLC-ICP-MS [64]. It became possible to separate several degradation products of organoarsenic warfare agents, such as phenylmethylarsinic acid (PMAA), phenyldimethylarsine oxide (PDMAO) and diphenylmethylarsine oxide (DPMAO), in the presence of DPAA and PAA. One of the analysed matrices was urine, which contributed to the broadening of knowledge on the metabolism of organoarsenic compounds in the body because several unknown organoarsenic compounds were found in addition to AB. Analyses were also conducted on rice and groundwater samples. Groundwater was collected from a well of about 5–6 m depth in Kamisu City, Japan. The analysis methods used in the team’s previous work did not yield satisfactory results, so it was decided to improve the research. However, a DRC module with oxygen as the reaction gas was used, and the monitored ion was AsO^+^ (*m*/*z* 91).

Several HPLC columns were verified: Inertsil C4 (150 mm × 2.1 mm, 5 µm); Inertsil NH2 (250 mm × 1.5 mm, 5 µm); Inertsil AS (150 mm × 2.1 mm, 3 µm) and guard columns, e.g., Inertsil C4 (10 mm × 1.5 mm, 5 µm); and Inertsil NH2 (10 mm × 1.5 mm, 5 µm). Patient urine samples were stored at −84 °C. The samples were diluted threefold for analysis and passed through a 0.45 μm membrane filter. The best separation of analytes was obtained on the Inertsil AS column, which was used to separate eight arsenic compounds, including MMA(V), DMAA, TMAO, TMA, AB and ACh. The next series of samples consisted of environmental samples.

Groundwater samples were diluted to ten times the original volume and filtered. Rice samples for analysis were prepared by pulverizing. Then, 0.2 g of powdered rice samples were weighed and extracted with 5 mL of methanol/water (1:1) solution at 80 °C. The obtained extracts were dried under reduced pressure, brought to 50 μL with water, and filtered. The samples were analysed by HPLC-ICP-MS. The final series of samples included urine samples taken from mice that had been given specially prepared contaminated food. Contamination included the addition of PAA to food, which was the main source of contaminants found in groundwater samples, and PMAA. The compound was found most abundantly in rice samples at a concentration of 1 μg/g of food. The samples were diluted twofold, filtered, and then analysed by LC-ICP-MS. The selected parameters for individual analyses are presented in Table 4.

The results of this study revealed that after oral administration to mice, PAA does not undergo metabolic changes (approximately 91% of the arsenic compounds in the analysed samples were PAA, in addition to PMAA and trace amounts of PDMAO). PMAA undergoes metabolic processes at approx. 12%. When the mice were contaminated with DPAA, no DPMAO was detected in the urine samples, but DPAA was present in the urine. This compound must have come from a different source, as it was also present in the blank sample.

It has also been proven that the unknown compounds seen in the chromatogram after analysing human urine samples were not the same as the peaks from the unknown arsenates in mouse urine samples. Therefore, it was likely that the arsenic compounds in the patient’s urine did not come from metabolites but from contaminated water, meaning that the types of arsenic compounds found in the patient’s urine depended on the exposure conditions. Unidentified compounds may also prove the complexity of metabolic processes in living organisms. It also follows that the metabolic processes of phenylarsenic compounds differ from those of inorganic arsenic compounds and that arsenic compounds containing the same hydroxyl groups do not necessarily follow the same metabolic processes. This difference may be due to the water solubility of the phenylarsenic compounds. PAA and DPAA are slightly soluble, whereas PMAA is very soluble. Kinoshita et al. demonstrated the importance of assessing the risk of exposure to arsenic under different conditions to obtain accurate results from analysing biological samples [61,62]. Rapid speciation analysis is particularly important for monitoring the content of organic arsenic in blood and urine, which allows for the verification of the presence of arsenic CWA residues.

GC-MS analyses suffer from the disadvantage that it is impossible to analyse organic and inorganic arsenic compounds simultaneously. The research of Daus et al. [65] aimed to develop an HPLC-ICP-MS method for determining organic (CWA derivatives) and inorganic arsenic compounds in groundwater samples during a single chromatographic analysis. In addition, the authors focused on a significant relationship, namely the high content of iron(II) in groundwater, which, after contact with atmospheric air, results in sediment precipitation in the sample and leads to the loss of some of the analytes. To preserve the sample, the researchers suggest adding phosphoric acid to a final concentration of 10 mM as soon as possible after collection. The validity of such a process has been confirmed by prior research [65]. The samples were protected at 6 °C for no longer than one week. The proposed method, which was based on the Shodex RSpack NN-614 column, was used to separate arsenic(III), arsenic(V), phenylarsenic acid, phenylarsinic oxide and diphenylarsenic acid (PAA, PAO and DPAA). The analysis time was 20 min. The highest DPAA contamination of groundwater was found at a depth of 2–3 m (3.505 mg/L), and the lowest was found at a depth of 14–15 m (0.019 mg/L). Inorganic arsenic compounds with a total content of up to 240 μg/L were also detected. Because of the possibilities offered by HPLC-ICP-MS, the simultaneous determination of organic and inorganic arsenic compounds was carried out, with an uncomplicated and quick sample preparation process, obtaining satisfactory results due to the low LOD of the method [65].

The team of Stetson et al. published the results of research on similar topics [66]. A method for separating four arsenic compounds, As(III), As(V), DMA and MMA, was described using HPLC-ICP-QQQ. The matrix included surface water and groundwater. The implemented method resulted in good separation of the compounds within 12 min. To preserve the samples, EDTA was added to each sample at a concentration of 2.5 mM. EDTA helped to reduce the redox reactions of arsenic compounds in the presence of iron, which was present in relatively high concentrations in groundwater samples. In addition, the samples were stored in the dark at 4 ± 2 °C. By using the proposed method, unprecedented detection limits were achieved: LOD_As(III)_ = 0.03 ng/mL, LOD_As(V)_ = 0.05 ng/mL, LOD_DMA_ = 0.03 ng/mL, and LOD_MMA_ = 0.04 ng/mL [66].

Hisatomi et al. published a research study on the possible metabolic pathways of arsenic compounds [67]. This study aimed to investigate the metabolic pathway and determine the main DPAA metabolite under anaerobic sulfate-reducing soil conditions. To study the metabolic pathway of arsenic compounds under such conditions, a soil culture was created with 30 mL of deionized water and 10.7 μg of As in the form of DPAA, 3.5 mg carbon (C)/g dry soil in the form of rice straw, and 425 μg sulfur (S)/g dry soil as K_2_SO_4_. The samples thus obtained were kept sealed in the dark at 30 °C for 3 weeks. After this, the samples were analysed. The chromatograms revealed a peak corresponding to a compound of unknown origin, with a concentration higher than the DPAA concentration calculated based on the peak intensity [67].

Using HPLC-ICP-MS, the unidentified arsenic compound formed in the reaction of DPAA with hydrogen sulfide showed the same retention time as the unknown compound, which was the product of metabolic changes in soil culture. Because of the intensity of the peak of the unknown compound and its absence in the analyses of soil culture samples without sources of carbon (C) and sulfur (S), as well as the inconsistency with the retention times of the peaks from As(V), PAA, PMAA and DPMAO, the authors concluded that this compound was diphenylthioarsinic acid (DPTA), the major metabolite of DPAA degradation in anaerobic and sulfur-reducing soil conditions [67]. The possible dimerization of this compound has also been proven.

The metabolic pathway by which most of the arsenic that enters the body is excreted in the urine as DMAA still needs to be fully understood. Suzuki’s team attempted to determine the ongoing transformations of organoarsenic compounds [68].

In the analysis of rat liver using HPLC-ICP-MS, several different organoarsenic compounds containing sulfur in their structure were discovered, including two unidentified compounds: dimethyldithioarsinic acid (dimethylarsinodithioic acid) (DMTA(V)) and dimethylthioarsinous acid (DMTA(III)). The rats used for the study were injected with arsenic compounds (DMAA, DMA(III), MMA(III) or MMA(V)) at a single dose of 0.5 mg As/kg body weight via the portal vein under anaesthesia. After 5 min, the rats were sacrificed, and their livers were excised for examination after whole-body perfusion. Arsenic solutions were adjusted to 1.0 mL/kg body weight [68].

For HPLC-ICP-MS analysis, 20 µL of the sample mixture was used, which was applied to a polymer-based gel filtration column (Shodex Asahipak GS-220 HQ, 300 mm 7.6 mm I.D., exclusion limit > 3000) or anion exchange column (Shodex Asahipak ES-502 N 7C, 100 mm, 7.6 mm I.D.). The column was eluted with 50 mM ammonium acetate buffer (pH 6.5 at 25 °C) or 15 mM citric acid buffer (pH 2.0 at 19 °C) using gel filtration or an anion exchange column at a flow rate of 0.6 or 1.0 mL/min. The outlet of the HPLC system was connected directly (with a PEEK tube 300 mm long, 0.25 mm I.D.) to the inlet of the ICP nebulizer. The *m*/*z* 75 (As^+^) and *m*/*z* 77 signals were monitored to compensate for ArCl^+^ molecular interference. The online ICP-MS data were processed by software developed in-house (Chiba University, Chiba, Japan) [68]. Liver samples were prepared by homogenization in four volumes of extraction buffer (50 mM ammonium acetate buffer, pH = 7.4) using a glass-Teflon homogenizer under nitrogen. The homogenates were centrifuged for 60 min at 4 °C to obtain the supernatant fraction. Low-molecular-weight arsenic compounds were well separated during the 30-minute analysis. Research has established that DMA(III) (and not DMAA) was converted to DMTA(V) and DMTA(III) in the liver supernatant. DMTA(V) could be present when the concentration of DMA(III) relative to the concentration in the liver supernatant was high [67,68].

A good understanding of the metabolism of organoarsenic compounds may contribute to the development of science in the context of a new decontamination approach.

In the study of Hempel et al. [56], the authors verified for the first time the influence of natural conditions on the degradation of persistent phenylarsenic compounds in groundwater under anaerobic conditions. This research aimed to investigate the potential of colonies of bacteria naturally occurring at sites contaminated with organoarsenic compounds to degrade PAA and DPAA under anaerobic conditions in situ.

Various organic and inorganic forms of arsenic have been detected and quantified by the speciation method described by Daus et al. using HPLC-ICP-MS with an RSpak NN-14 column (150 × 6 mm, Shodex, Tokyo, Japan) [65].

The dominant forms/compounds of arsenic in the original sample were As(V), PAA and DPAA. The total concentration of arsenic was 1755 μg/L. In living microcosms, after 13 weeks of incubation, more than 95% of the PAA had been removed from the solution. The total arsenic content showed a similar trend, with an overall elimination rate of approximately 77%. In contrast to the concentration of PAA, the concentration of DPAA was stable throughout the cultivation period in both the live and lactated environments. During the first and fourth weeks of culture, an unknown organoarsenic compound was detected as an intermediate. In the living microcosms enriched with lactate, the PAA concentration decreased by approximately 98% within five weeks. PAO formed after 14 days and was removed after 7 weeks. Like unknown arsenic compounds in living environments, PAO is a metabolite in lactate-enriched cultures during the PAA transformation process. The total arsenic concentration was reduced to 81% of the initial concentration with the addition of lactate [65].

Because arsenic compounds are chemically stable under sterile conditions, the observed transformations of arsenic compounds in living microcosms are believed to result from microbial activity and the resulting reducing conditions. The faster reaction rate in the presence of lactate can be explained by the lower redox potential in these samples and/or the higher concentration of sulfides in the water. Studies have shown that the concentration of organic arsenic compounds in anaerobic groundwater samples can be significantly reduced through a process mediated by bacteria. This article also presents probable structural formulas of unknown metabolites of organoarsenic compounds [65].

## 5. Concluding Remarks

As demonstrated, the HPLC-ICP-MS technique allows for the rapid detection of trace concentrations (ppb level) of chemical warfare agents and their degradation products. In Table 5, we summarized the selected analytes and the corresponding detection limits published in research articles [6,33,52,63,64,69,70,71]. Each HPLC-ICP-MS analysis should consider several possible interferences related to the same mass of different ions.

The modernization of ICP-MS technology in the form of a collision reaction cell, or rather one of its types, ORS, allows for the elimination of these disturbances.

A fast analysis that does not require complicated sample preparation procedures may be crucial in the face of the growing threat of using chemical warfare agents and other highly toxic substances during wars, terrorist attacks, sabotage, and/or chemical accidents.

HPLC-ICP-MS can be used as a complement to currently commonly used GC-MS and HPLC-MS techniques for the determination of CWAs and their degradation products in environmental and biological samples. In special cases, it may be the leading analytical technique.

A relatively small number of research papers describing the use of HPLC/GC-ICP-MS show the still unexplored possibilities of this analytical tool, and the documented results of analyses of CWAs in composed samples indicate its great potential. HPLC-ICP-MS can also be used to verify the provisions of the Chemical Weapons Convention (CWC) [72]. We believe that the use of the HPLC-ICP-MS technique is currently underestimated in studies of the presence of CWAs and their metabolic products in living organisms and the environment. The increasing availability and popularity of the HPLC-ICP-MS technique allow us to conclude that it will be used more frequently in CWC and Homeland Security analytical laboratories in the future.

Equipping laboratories with HPLC-ICP-MS is justified and will increase the possibility, credibility and reliability of laboratory analyses of CWAs, their derivatives and other toxic and hazardous substances in environmental and biological samples.

## Figures and Tables

**Table 1:** Comparison of the detection limits (LODs) for HPLC-ICP-MS and GC-ICP-MS [31,33].

Analyte	LOD for HPLC-ICP-MS [pg/mL]	LOD for GC-ICP-MS [pg/mL]
EMPA	263	34.2
IMPA	183	20.9
MPA	139	49.6

**Table 2:** Some parameters for the separation of OPNA DPs [34,35].

No.	Type of Column	Mobile Phase	Flow [mL/min]	V_injection_ [μL]	Analytes	LOD [ng/mL]	Separation Time [min]
1.	Dionex IonPac AS7 4.1 mm × 250 mm, guard column	0.4 mM acetic acid/sodium acetate, 5 mM HNO_3_ (pH∼2.4) in DDW	1.0	25	EMPA	d.n.a.	5
					IMPA	d.n.a.	
					MPA	d.n.a.	
2.	Hamilton PRP-X100 4.6 mm × 100 mm 5 μm	A = 0.5% H_2_CO_3_ (pH∼2.3), 5% MeOH in DDW B = 0.3 M NH_4_HCO_3_ (pH∼2.3), 22% MeOH in DDW	1.0	100	EDHAP	21.7	25
					MPA	18.3	
					EPA	19.9	
					DMHP	10.0	
					PPA	22.9	
					EMPA	20.4	
					IMPA	19.5	
					DEHP	26.6	
					IPHEP	61.5	
					IBHMP	81.1	
3.	Hamilton PRP-X100 2.1 mm× 150 mm 5 μm	A = 10 mM (NH_4_)_2_CO_3_ (pH∼8.5) in DDW B = 50 mM (NH_4_)_2_CO_3_ (pH∼8.5) in DDW	0.5	100	EDHAP	88.1	30
					MPA	7.4	
					EPA	7.4	
					DMHP	8.9	
					PPA	14.5	
					EMPA	11.2	
					IMPA	28.6	

d.n.a.—Data not available.

**Table 3:** Selected parameters of the determinations carried out in the work of Kroening et al. [52].

STRUCTURE	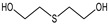	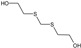	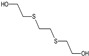	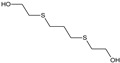	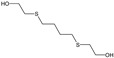
ACRONYM	TDG	BHETM	BHETE	BHETPr	BHETBu
COMPOUND NAME	Thiodiglycol	Bis(2-hydroxyethylthio)methane	1,2-bis(2-hydroxyethylthio)ethane	1,3-bis(2-hydroxyethylthio)propane	1,4-bis(2-hydroxyethylthio)butane
LOD [NG/ML] STUDY	4.60	35.50	79.30	98.50	73.20
LOQ [NG/ML] RIVER WATER SAMPLES	750	690	1000	1160	3300

**Table 4:** Summary of analysis parameters and the obtained limit of detection in the work of Kinoshita et al. [63,64].

Publication [64]					Publication [63]
Mechanism		Hydrophobicity	Dipole–Dipole Interaction	Separation of Functional Groups	
Column		C_4_	CN	NH_2_	C_8_
Organic solvents		H_2_O (HNO_3_)/C_2_H_5_OH/ACN = 80:15:5,	citrate buffer/CH_3_OH/ACN = 70:20:10,	phosphate buffer/ACN = 50:50	0.1% HCOOH–CH_3_CN = 80:20
pH		1.5	5.5	2.5	2.0
T_column_ [°C]		40	40	40	40
Flow [mL/min]		0.3	0.3	0.1	0.2
V_injection_ [μL]		20	10	no satisfactory separation; the NH_2_ column was not used for further research	20
LOD [ng/mL As]	PAA	0.25	1.0		0.3
	PMAA	0.25	0.5		-
	DPAA	0.5	-		0.5
	PDMAO	0.1	0.5		-
	DMPAO	0.3	1.0		-

**Table 5:** Comparison of the detection limits (LODs) of HPLC-MS-MS and HPLC-ICP-MS based on selected methods in which the CWA degradation products were analytes [6,33,52,63,64,69,70,71].

No.	Analyte	LOD [ppb = ng/mL]			
		HPLC-MS/MS	Ref.	HPLC-ICP-MS	Ref.
1.	MPA	10	[69]	0.14 ^(a)^	[33]
2.	EMPA	5 * 1 **	[70]	0.03 ^(a)^	[33]
3.	IMPA	5 * 1 **	[70]	0.02 ^(a)^	[33]
4.	TDG	40	[71]	4.6	[52]
5.	CVAOA	500	[6]	0.1	[63]
6.	PAA	50	[6]	0.25	[64]
7.	PMAA	1	[6]	0.25	[64]

* (SIM mode), ** (SRM mode), ^(a)^ approximate value to the second decimal place.

## Abbreviations and Acronyms

**Acronym and Abbreviation**	**Full Name**	**No CAS**
**AB**	Arsenobetaine	64436-13-1
**ACh**	Acetylcholine	51-84-3
**AChE**	Acetylcholinesterase	9000-81-1
**APCI**	Atmospheric Pressure Chemical Ionization	-
**BCVAA**	Bis(2-chlorovinyl)arsinous acid	NF
**BDPAO**	Bis(diphenylarsine)oxide	2215-16-9
**BHETBu**	1,4-bis(2-hydroxyethylthio)butane	7425-93-6
**BHETE**	1,2-bis(2-hydroxyethylthio)ethane	5244-34-8
**BHETM**	Bis(2-hydroxyethylthio)methane	44860-68-6
**BHETPr**	1,3-bis(2-hydroxyethylthio)propane	16260-48-3
**CCT**	Collision-reaction Cell Technology	-
**CMPA**	Cyclohexyl methylphosphonic acid	1932-60-1
**CVAA**	2-chlorovinylarsenous acid	85090-33-1
**CVAO**	2-chlorovinylarsine oxide	3088-37-7
**CVAOA**	2-chlorovinylarsonic acid	64038-44-4
**CWA**	Chemical Warfare Agents	-
**CWC**	Chemical Weapons Convention	-
**DEHP**	Diethylhydrogen phosphate	598-02-7
**DMA (III)**	Dimethylarsinous acid	55094-22-9
**DMAA**	Dimethylarsinic acid	75-60-5
**DMHP**	Dimethylhydrogen phosphate	813-78-5
**DMTA (III)**	Dimethylthioarsinous acid	NF
**DMTA (V)**	Dimethyldithioarsinic acid (dimethylarsinodithioic acid)	NF
**DPAA**	Diphenylarsinic acid	6217-24-9
**DPCA**	Diphenylchloroarsine	712-48-1
**DPCNA**	Diphenylcyanoarsine	23525-22-6
**DPMAO**	Diphenylmethylarsine oxide	NF
**DPTA**	Diphenylthioarsinic acid	NF
**DRC**	Dynamic reaction cell	-
**EDTA**	Ethylenediaminetetraacetic acid	60-00-4
**EHDAP**	Ethyl hydrogen dimethylamidophosphate sodium salt	NF
**EMPA**	Ethyl methylphosphonic acid	1832-53-7
**EMPTA**	Ethyl methylphosphonothioic acid	18005-40-8
**EPA**	Ethylphosphonic acid	6779-09-5
**EPCN**	Ethylphosphorylcyanidate	NF
**ESI**	Electrospray ionization	-
**GA**	O-Ethyl N,N-dimethyl phosphoramidocyanidate (Tabun)	77-81-6
**GB**	Propan-2-yl methylphosphonofluoridate (Sarin)	107-44-8
**GC**	Gas Chromatography	-
**GD**	3,3-Dimethylbutan-2-yl methylphosphonofluoridate (Soman)	96-64-0
**GF**	Cyclohexyl methylphosphonofluoridate (Cyclosarin)	329-99-7
**GFAAS**	Graphite Furnace Atomic Absorption Spectroscopy	-
**HD**	bis(2-chloroethyl)sulfide (Sulfur mustard)	505-60-2
**HPLC**	High-Performance Liquid Chromatography	-
**IBHMP**	Isobutyl hydrogen methylphosphonate	1604-38-2
**IC_50_**	Median inhibitory concentration	-
**ICP-MS**	Inductively Coupled Plasma-Mass Spectrometry	-
**IMPA**	Isopropyl methylphosphonic acid	1832-54-8
**IPHEP**	Isopropyl hydrogen ethylphosphonate	170135-50-9
**LC**	Liquid Chromatography	-
**LOD**	Limit of detection	-
**MMA (III)**	Monomethylarsonous acid	25400-23-1
**MMA (V)**	Monomethylarsonic acid	124-58-3
**MPA**	Methylphosphonic acid	993-13-5
**MS**	Mass spectrometry	-
**OES**	Optical emission spectroscopy	-
**OPNA**	Organophosphorus Nerve Agents	-
**OPNA DP**	Organophosphorus Nerve Agents Degradation Products	-
**ORS**	Octopole Reaction System	-
**PAA**	Phenylarsonic acid	98-05-5
**PAO**	Phenylarsine oxide	637-03-6
**PDCA**	Phenyldichloroarsine	696-28-6
**PDMAO**	Phenyldimethylarsine oxide	NF
**PMAA**	Phenylmethylarsinic acid	NF
**PMPA**	Pinacolyl methylphosphonic acid	616-52-4
**PPA**	Propylphosphonic acid	4672-38-2
**ppb**	Parts per billion	-
**ppm**	Parts per million	-
**ppt**	Parts per trillion	-
**PTE**	Phosphotriesterase	-
**QQQ**	Triple quadrupole (mass spectrometer)	-
**TBDMS**	Tert-Butyldimethylsilyl ether	124150-87-4
**TDG**	Thiodiglycol	111-48-8
**TMA**	Tetramethylarsonium	NF
**TMAO**	Trimethylarsine oxide	4964-14-1
**UV**	Ultraviolet	-
